# Medication Interaction and Physicians' Compliance Assessment through Medication Reconciliation Forms in a University Affiliated Hospital

**Published:** 2018

**Authors:** Mohammad Mehdi Talebi, Aida Sefidani Forough, Parsa Riazi Esfahani, Raha Eskandari, Roodabeh Haghgoo, Fanak Fahimi

**Affiliations:** a *Department of Clinical Pharmacy, School of Pharmacy, Shahid Beheshti University of Medical Sciences, Tehran, Iran. *; b *Irvine Valley College, Irvine, CA, USA.Tehran, Iran.*

**Keywords:** Adverse drug reaction, Medication discrepancies, Medication reconciliation, Medication error, Drug interactions

## Abstract

Medication interactions are associated with various unwanted adverse drug reactions. Medication Reconciliation involves a process in which a complete list of patient's previously prescribed medications are recorded and subsequently evaluated within the context of concomitantly prescribed medications and present medical condition during the hospitalization. Medical records of randomly selected 270 patients hospitalized in internal medicine, cardiovascular and infectious diseases wards were evaluated. Drug interactions were checked by LexiComp® database. Each interaction was assigned a risk rating of A, B, C, D, or X. The progression from A to X was based on increased urgency for responding to the data. Completed reconciliation forms were attached to patient charts for evaluation of physicians' compliance. Drug interactions were observed in 65.2% (176/270) of cases. The risk rating of interactions was categorized as C, D and X in 54.2%, 32.4%, and 13.4% of cases, respectively. There was a positive correlation between the number of prescribed medications and the rate of interactions (*p*-value < 0.001, Kendall's correlation coefficient = 0.487). Moreover, the length of hospitalization and the rate of drug interactions were significantly correlated (*p*-value < 0.001, Kendall's correlation coefficient = 0.350). Cardiovascular agents constituted the largest proportion of interactions (25%) followed by antibiotics (18%) and immunosuppressive agents (6%). In 59.6% of cases, no corrective action was taken by the physicians. Medication discrepancies occur commonly in hospital settings. Structured medication reconciliation may have a positive impact on prevention of medication errors.

## Introduction

Preventable medication errors are associated with considerable rate of mortality or patient harm as well as a significant economic burden on health care system. One Research shows that approximately 7000 deaths out of overall 44000-98000 deaths associated with errors in medical care are due to medication errors (1).

Drug interactions which are one of the main subsets of medication errors may also cause various unwanted adverse reactions (1).

The risk and severity of drug interactions depends on different factors including the number of prescribed medications, duration of treatment, patient's age, and the stage of the disease (2). Results of a recent study indicated that 72 percent of medication discrepancies occur due to errors in medication history taking during patients' hospital admission, while 26 percent stem from lack of appropriate reconciliation of drug history with discharge orders (3).

Pharmacists play an important role in prevention, detection and management of medication errors. The ultimate goal of clinical pharmacy is to optimize the prescription, administration and use of medications which can be obtained by maximizing the therapeutic effects of drugs, choosing the best treatment strategy for different diseases, reducing the risk of adverse drug events, therapeutic drug monitoring (TDM), introducing alternative therapies when necessary and collaborating with patients during their treatment course. These services are provided in three levels of before, during, or post administration.

Medication Reconciliation is defined as a process in which a complete list of patient's previously prescribed medications are recorded at the time of hospital admission or any other transfer and subsequently are compared within the context of concomitantly prescribed medications and present medical condition during the hospitalization (4). Therefore, medication reconciliation can be used as a strategy to prevent medication errors and assist decision making in prescribing drugs by identifying existing discrepancies in pharmacotherapy regimens in various healthcare settings (5).

Pharmaceutical care departments in hospitals play a crucial part in this regard as all drug- related issues and pharmaceutical education and research programs in hospitals directly controlled by these departments (6). Successful medication reconciliation programs are mainly implemented by pharmacists. A systematic review indicated that 85 percent of medication reconciliations were performed by these healthcare professionals. (7). It has been suggested that documentation of drug histories can be more effective when they are acquired by pharmacists (8). Also, patient allergies are documented more accurately when pharmacists are involved (9).

As shown by a study, a set of pharmacist interventions during a 7-month course not only led to a 70% decline in overall rate of medication errors, but also reduced the incidence of adverse drug reactions by 15 % (10). It has been shown by a recent study that pharmacist intervention can also affect physicians' decisions in terms of appropriate medication dose adjustment and early reaction time (11). Moreover, results of another study indicated that pharmacist involvement from the beginning of the medication reconciliation program led to a considerable decrease of 80% in total adverse drug reactions encountered during a 3-month investigation (12). A successful medication reconciliation program may reduce the duration of time allocated for medical care of each patient as well (13). A recent study by Kwan *et al* which focused on the economic impact of medication reconciliation program revealed that even one percent improvement in prevention of clinically significant medication interactions may reduce the indirect healthcare costs mainly associated with increased length of hospitalization considerably each year (14).

The importance of the subject could be even greater in developing countries like Iran, where therapeutic monitoring such as blood level monitoring is rarely performed in medical settings (15). In this study we aimed to evaluate the medication interactions using medication reconciliation forms in internal medicine, cardiovascular and infectious disease wards in Masih Daneshvari Hospital, Tehran. In addition, we attempted to categorize these interactions based on their severity and to find any correlation between the number of prescribed medications and the length of hospitalization with the rate of medication errors.

## Experimental

This was a cross sectional observational study, which was conducted in a 11 month period from April 2014 to July 2015 in Masih Daneshvari Hospital, a tertiary university affiliated center.

Medical kardexes and charts of 270 patients hospitalized in internal medicine, cardiovascular and infectious disease wards were randomly selected for assessment. We used a pre-designed structured medication reconciliation form as our data collection instrument. These forms consisted of different sections including patient demographics, a complete list of patient’s drug history (e.g. name, dose, route of administration, dosing intervals) and also a Table for patient’s current medication profile, detected medication errors, and any record of corrective action by healthcare professionals if any interaction existed (i.e. drug discontinuation, order modification or no change).

Patients’ drug history was obtained through previous prescriptions, patients’ drug bag, family members and the drug history which was recorded by the nurses at the time of hospital admission.

All forms were then assessed using LexiComp® software for drug interactions. The filled medication reconciliation forms were attached to patient’s chart for physician’s review and their compliance was assessed subsequently.

In LexiComp® database one separate monograph has been introduced for each drug-drug interaction which classifies them based on their risk rating, severity, and documentation reliability rating (16). Recommendations for preventing adverse outcomes resulting from an anticipated drug interaction and a brief presentation of published data are also available in this program. 

In terms of risk rating assessment, each interaction monograph is assigned a degree labeled by A, B, C, D, or X. The progression from A to X is accompanied by the increased urgency of responding to the data and is mainly an indicator of the clinical significance of the interaction (17). Severity rating on the other hand, refers to the extent of the pharmacokinetic and pharmacodynamic interactions and is categorized in three levels of severe, moderate, and mild interaction. 

The Reliability Rating provides an indication regarding the volume and quality of reports used to create the interaction monograph. Ratings include EXCELLENT (multiple RCTs; OR single RCT plus > 2 case reports), GOOD (single RCT plus < 2 case reports), FAIR (documentation in the prescribing information only, or > 2 case reports; OR < 2 case reports plus other supporting data; additional descriptors note whether the supporting data are inconsistent or have no known mechanistic basis), POOR (< 2 case reports with no other supporting data), or THEORETICAL (no published clinical documentation, but interaction based on known or predictive pharmacology). 

In order to have the same chance for each variable when evaluating the rate of drug interactions among different drug categories, we used a correction factor which means an interaction being evaluated for the same number of encounters.

Statistical analysis was performed by SPSS version 22.0. If normally distributed, we used Student’s t-test, Mann- Whithney non-parametric test Kruskal- Wallis analysis in our statistical analysis. Comparison of two groups of qualitative data was performed using Pearson Chi-square analysis or Fisher’s exact test when required. Moreover, Kendall correlation coefficient was used to assess the association between two measured quantities as they were not normally distributed. In order to assess the possibility of medication interaction occurrence in different groups, we used Logistic Regression. A *p*-value of < 0.05 was considered statistically significant.

## Results

A total of 270 patient records were evaluated. The mean age of patients was 55.5 ± 17.9 years (Max 91, Min 13). Male: female ratio was 159:111. Regarding the medical history of evaluated patients, cardiovascular diseases were the mainly encountered pre-existing conditions (30.2%) followed by smoking and opioid use (21.1%) while HIV infection was the least encountered condition among patients (0.2%) (Table 1).

Patients’ drug history records indicated that a total number of 1136 medications had been prescribed before their hospital admission. Cardiovascular medications constituted the largest proportion in patients’ drug history (33.0%) (Table 2).

On the other hand, the number of prescribed medications during hospital stay was found to be 2697, among which cardiovascular agents and antibiotics had the highest rate of prescription with 19.9% and 15.0%, respectively. Atorvastatin- pantoprazole co-administration constituted the highest rate of interactions with 38 cases.

The mean number of medication per patient was 9.73 ± 4.10.

At least one interaction was detected in 176 (65.2%) patient charts and totally 426 interactions were observed. 

Statistical analysis showed a significant positive correlation between patient age and number of drug interactions (*p*-value < 0.05).

Moreover, a positive correlation was identified between the number of prescribed medications and the rate of interactions (*p*-value < 0.001, Kendall's correlation coefficient = 0.487) (Table 3) as well as between the length of hospitalization and the number of drug interactions (*p*-value < 0.001, Kendall's correlation coefficient = 0.350) (Table 4). However, there was no gender-related difference for medication interactions.

In terms of drug categories with the most interactions, cardiovascular agents were responsible for 25% of the total interactions. Details of the interaction rate for each drug category along with the selected drug from each class with the highest interaction incidence are presented in Table 5. and Table 6 illustrates the Figures for the rate of interactions before and after correction factor in each ward.

Risk rating assessment results indicated that category X, D, and C interactions were in ascending order of 13.4%., 32.4% and 54.2%. Additionally, considerable rates of 180 (42.3%) interactions were categorized as severe. This was followed by 239 (56.1%) moderate and 7 (1.6%) mild interaction cases. 

Regarding the reliability rating of interactions only 26 (6.1%) of interaction documentations were categorized as excellent while the majority of documentations were either good (28.0%) or fair (64.5%). Poor reliability was also detected for 6 (1.4%) interactions.

Looking at the rates of interactions in different wards, we found that drug interactions were highly prevalent in Internal medicine Ward 3 (27%). Contrarily, CCU_2 _and Post-CCU showed a better profile of drug interactions with only 5% of the overall interactions. However, after applying correction factor Post-CCU constituted the largest proportion of drug interactions among all wards with 16.7%. In terms of patient length of stay, CCU_1_ and CCU_2_ had the highest and lowest hospitalization days, respectively (15.15 vs. 8.55).

In terms of complications that were possible to arise from our recorded interactions, QT prolongation and arrhythmia had the largest proportion (27.5%) followed by myopathy (12.5%). Enhanced anti-cholinergic effects, serotonin syndrome, increased or decreased rate of absorption and increased risk of neurotoxicity were among the other potential consequences of recorded drug interactions.

In 94 (34.8%) cases no interactions were found. Changing the time of administration, and/or interval was performed by informing the responsible nurse to prevent 15 (5.6%) potential interactions in patients’ medication orders. In 161 (59.6%) forms of all 270 medication reconciliation forms no corrective action was taken by the physicians.

## Discussion

The present study which was performed on patients in cardiology, internal medicine and infectious diseases wards of Masih Daneshvari Hospital, revealed that while the overall encountered drug interactions were 426 cases, these interactions encompassed almost two-third of the studied population who had the potential of drug interactions. According to our study reference, 42.3% of interactions were classified as severe while the main proportion of interactions (56.1%) was moderate. This high rate of potential drug interactions regardless of their actual occurrence should be scrutinized very closely by healthcare professionals. Providing these professionals with competent knowledge about different types and mechanisms of drug interactions along with prevention strategies for drug interactions can be of great importance. 

**Table 1:** Pre-existing medical conditions of patients

**Pre-existing medical condition**	**Number**	**Percent**
Cardiovascular Disease	169	30.2
Smoking/Opium Use	118	21.1
Respiratory Disease	64	11.4
Diabetes	45	8.0
Tuberculosis	37	6.6
Food/Drug Allergy	29	5.2
Psychiatric Disease	13	2.3
Thyroid Disease	10	1.8
Renal Disease	10	1.8
Alcohol Use	6	1.0
Hepatitis	2	0.4
AIDS	1	0.2
Other	56	10.0

**Table 2:** Drug Categories in Patients' Drug History

**Drug Category**	**Number**	**Percent**
Cardiovascular Agents	375	33.0
Bronchodilators	125	11.0
Antibiotics	118	10.4
Anticoagulants	90	8.0
Supplements	83	7.3
Gastrointestinal Agents	68	6.0
Anti-diabetics	58	5.1
Anti-coughs	47	4.1
Corticosteroids	41	3.6
Nervous System Agents	40	3.5
Analgesics	18	1.6
Immunosuppressants	15	1.3
Thyroid Agents	9	0.8
Sedatives	9	0.8
Antihistamines	8	0.7
Anti-neoplastic Agents	7	0.6
Anti-rheumatic Agents	7	0.6
Antigout Agents	6	0.5
Electrolytes	5	0.4
Antivirals	3	0.3
Antifungals	3	0.3
Herbal Medicines	1	0.1

**Table 3:** Association of the number of prescribed drugs with occurrence of drug interactions

		**Mean**	**SD**	***P*** **-value**
Overall Prescribed Medications	With interaction	11.26	3.60	<0.001
	Without interaction	6.87	3.41	

**Table 4:** Association of the duration of hospitalization with occurrence of drug interactions

		**Mean**	**SD**	***P*** **-value**
Days of Hospitalization	With interaction	14.84	10.20	<0.001
	Without interaction	8.21	5.59	

**Table 5.** Rate of drug interactions in each drug category

**Medication Category**	**Rate of interaction (%)**	**Medication with highest rate of interaction in each category (N)**
Cardiovascular	186 (24.6)	Atorvastatin (63)
Antibiotics	135 (17.9)	Ciprofloxacin (36)
Respiratory system	130 (17.2)	Combivent (41)
Sedatives, hypnotics and narcotics	99 (13.1)	Methadone (25)
Gastrointestinal	89 (11.8)	Pantoprazole (57)
Vitamins and supplements	67 (8.9)	Calcium-D (34)
Immunosuppressants and corticosteroids	49 (6.5)	Prednisolone (17)

**Table 6:** Rate of drug interactions in different wards of the hospital before and after applying correction factor.

**Ward**	**Number of observations**	**Rate of interactions before applying correction factor (%)**	**Rate of interactions after applying correction factor (%)**
Internal 3	47	35 (20)	74.5 (13.8)
Internal 4	68	47 (27)	69.1 (12.8)
Infectious Diseases Ward 5	31	17 (9.5)	54.8 (10.2)
Infectious Diseases Ward 6	44	22 (12.5)	50.0 (9.3)
Internal 9	37	26 (15)	70.3 (13.1)
CCU_1_	13	11 (6)	84.6 (15.7)
CCU_2_	20	9 (5)	45.0 (8.4)
Post-CCU	10	9 (5)	90.0 (16.7)
Total	270	176 (100)	538.3 (100)

Comparison of drug interaction studies has some limitations due to different factors. Differences in study designs, methods and definitions lead to significant variation in the incidence of reported drug interactions (18). Also, some researchers base their results on theoretical aspects of the interaction while their counterparts may only consider the clinical features of the interactions in their judgments. 

Considering these facts, the comparison of medication interaction related studies in Iran indicates an ascending trend in the rate of drug interactions (19, 20, 21 and 22). As shown by local studies, Iran has a comparatively higher rate of interactions than developed countries like the United States or France (23, 24 and 25) and our results are also in concordance with these previous findings. High number of drugs per prescription in Iran (26, 27) can be the leading cause of this relatively elevated rate of interactions.

In 2005, Nazari *et al.* found similar results in an interaction investigation in ICU (28). Another study which investigated the drug use patterns in ICU indicated a positive correlation between overall prescribed drugs and antibiotics and patients' mortality (29).

A majority of patients in our study were over 60 years of age who were at considerably higher risk of drug interactions compared to young patients. Older patients become more prone and vulnerable to drug interactions due to various co-morbidities, more severe health conditions (30) as well as physiologic changes (31). Considering the fact that the use of cardiovascular agents is very common in these population, the findings of our study indicated that not only the highest rate of drug interactions belong to cardiovascular drugs but also Post-CCU ward had the highest rate for these interactions. Patients are prescribed a lot of medications for their cardiovascular and other concomitant illnesses in this ward. Therefore, the potential of drug interaction occurrence is relatively high since the majority of patients are elderly people with polymedication whose drug metabolism has altered due to existing cardiac disease (32).

We also tried to investigate whether there was a correlation between the number of interactions with duration of hospital stay. Expectedly, we found a positive correlation between these two variables (*p*-value < 0.001, Kendall's correlation coefficient = 0.350). This can be explained by assuming that patients with more serious health conditions, spend longer in hospitals where they are prescribed with different pharmacotherapy regimens from various drug classes. Our findings are in accordance with a previous study by Classen *et al.* who reported that drug interactions directly influence the hospital length of stay, medical costs, and the risk of mortality (33). Conversely, Danielson et.al stated that longer hospital stay is associated with the higher risk of developing drug interaction in patients (34).

All hospitalized patients in our center received gastric ulcer prophylaxis mainly pantoprazole. On the other hand, atorvastatin is a frequently prescribed medication. Therefore, pantoprazole- atorvastatin, a PPI-cardiovascular agent co-administration was the most prevalent drug interaction observed during the study period. A similar study by Durrence *et al*. reported cimetidine and digoxin interaction responsible for 90 percent of severe drug interactions (34). Among complications that were possible to arise from our recorded interactions, QT prolongation and arrhythmia had the largest proportion (27.5%), followed by myopathy (12.5%). Approximately 10 percent of detected discrepancies were due to interactions that altered absorptions that could be easily prevented by changing the administration time or intervals of drugs. However, these interactions were either neglected or missed by physicians and therefore it necessitated the involvement of a pharmacist to reduce these errors. Concerning fact about our results was that, although all filled reconciliation forms were available in patient charts for physicians' review and despite consistent pharmaceutical care department follow ups, almost 60 percent of reconciliation forms were overlooked or disregarded by physicians. Established position of pharmacists in healthcare system is extremely needed for optimal patient care as drug interactions are chiefly detected and reported by pharmacists (35). Implementing a clinical pharmacy education program for pharmacy students and clinical pharmacy residents in a teaching hospital in Iran has shown successful outcomes (36). There are some strategies such as creating standardized drug administration charts, improving communication among healthcare professionals, preventive education and using information technology to improve medication safety (37).

Future improvements in this field need a continuous effort and cooperation among funders, regulators, health professionals, researchers and health services. Development of multidisciplinary processes and implementation of computerized health systems in healthcare settings plays an important role in this regard (38).

One of the limitations of this study is that, due to its single site design, the findings might not be extrapolated to other settings, therefore further multi-site studies are required. Moreover, in our assessment of physicians’ compliance we were not able to consider possible confounding factors such as physician’s clinical judgment or inadequate communication and collaboration among physicians, pharmacists and other healthcare professionals in the final decision. Therefore, further studies are needed to determine the underlying causes of this high rate of non-compliance. 

## Conclusion

Our study demonstrated a high rate of medication discrepancies among inpatients. Getting benefit from the services provided by professional pharmacists in hospital settings can facilitate medication reconciliation; however, more physician awareness is needed especially in developing countries like Iran.
